# Performance of a Mid-Infrared Sensor for Simultaneous Trace Detection of Atmospheric CO and N_2_O Based on PSO-KELM

**DOI:** 10.3389/fchem.2022.930766

**Published:** 2022-07-14

**Authors:** Guolin Li, Zecheng Zhang, Xuena Zhang, Yunhui Wu, Kun Ma, Yue Jiao, Hao Zhao, Yimeng Song, Yajing Liu, Shenqiang Zhai

**Affiliations:** ^1^ Key Laboratory of Laser Technology and Gas Sensing, College of Control Science and Engineering, Ministry of Education, China University of Petroleum (East China), Qingdao, China; ^2^ Institute of Semiconductors, Chinese Academy of Sciences, Beijing, China

**Keywords:** tunable diode laser absorption spectroscopy, wavelength modulation spectroscopy, quantum cascade laser, carbon monoxide, nitrous oxide

## Abstract

In this article, a field deployable sensor was developed using a self-developed 4.58-µm continuous wave quantum cascade laser (CW-QCL) for the simultaneous detection of carbon monoxide (CO) and nitrous oxide (N_2_O), both of which have strong fundamental absorption bands in this waveband. The sensor is based on tunable diode laser absorption spectroscopy (TDLAS) technology, which combined a multi-pass gas cell (MPGC) with a 41 m optical path length to achieve high-precision detection. Meanwhile, the particle swarm optimization-kernel extreme learning machine (PSO-KELM) algorithm was applied for CO and N_2_O concentration prediction. In addition, the self-designed board-level QCL driver circuit and harmonic signal demodulation circuit reduce the sensor cost and size. A series of validation experiments were conducted to verify the sensor performance, and experiments showed that the concentration prediction results of the PSO-KELM algorithm are better than those of the commonly used back propagation (BP) neural networks and partial least regression (PLS), with the smallest root mean square error (RMSE) and linear correlation coefficient closest to 1, which improves the detection precision of the sensor. The limit of detection (LoD) was assessed to be 0.25 parts per billion (ppb) for CO and 0.27 ppb for N_2_O at the averaging time of 24 and 38 s. Field deployment of the sensor was reported for simultaneous detection of CO and N_2_O in the air.

## Introduction

In recent years, the problem of global warming has become increasingly serious due to the massive emissions of greenhouse gases. N_2_O is an important greenhouse gas with a concentration of about 320 ppb in the troposphere under normal conditions, but it produces about 300 times the greenhouse effect of CO_2_ and has a lifetime in the atmosphere of up to 120 years (Montzka et al., 2011). The current high use of nitrogen fertilizers in global food production has led to a yearly increase in N_2_O emissions, with global anthropogenic N_2_O emissions having increased by 30% over the last 40 years; its large emissions seriously damage the ozone layer and exacerbate the development of global warming (Tian et al., 2020). At the same time, CO is also considered to be an important component of the atmosphere, mainly from vehicle emissions and incomplete combustion of hydrocarbons. With the development of human society, the concentration of CO in the troposphere has exceeded 100 ppb, which not only endangers human health but also reacts with other gases in the atmosphere to produce photochemical smog, seriously affecting the ecological environment (Thompson, 1992). Therefore, trace detection of atmospheric CO and N_2_O can determine the emissions of both in different regions at different times, which is important for studying the earth’s climate and environmental changes; it can also be used for environmental monitoring as a way to control anthropogenic CO and N_2_O emissions. That is because N_2_O from fossil fuel combustion can be significantly higher than that in natural situations.

Compared to traditional non-optical atmospheric trace gas detection methods such as electrochemical methods and gas chromatography (Tatsumi et al., 2017), spectroscopic detection techniques have been shown to have low LoD, high sensitivity, and fast response times for atmospheric trace gas detection. It mainly includes non-dispersive infrared (NDIR) (Wang et al., 2017), TDLAS, and photoacoustic spectroscopy (PAS), etc. Among them, TDLAS technology is very widely used in the field of atmospheric environment monitoring, which has the characteristics of high spectral resolution and high detection sensitivity and is very suitable for trace detection of atmospheric environment gases (Liu et al., 2020). Wavelength modulation spectroscopy (WMS) and direct absorption spectroscopy are two widely used variations of TDLAS. To improve the signal-to-noise ratio of the sensor to raise the lower LoD, WMS should be selected as the basic detection strategy of the sensor. Moreover, CO and N_2_O absorb more strongly in the mid-infrared waveband, and QCL has the advantages of narrow line width and high output optical power among mid-infrared laser light sources, so there are many reports choosing QCL combined with TDLAS technology for trace gas detection (Suchalkin et al., 2015; Hashimoto et al., 2017). For example, Nelson et al. (2004) used a 7.9-μm pulsed-wave (PW) QCL combined with a 56-m MPGC to detect atmospheric N_2_O and CH_4_ at low concentrations; Fritsch et al. (2007) developed a 4.97-μm QCL-based trace gas sensor and applied it to the life sciences for the detection of trace CO in human exhalation; Rice University’s Kohring et al. (2014) detected NO in biological samples using a 5.2-μm CW-QCL combined with a Herriot gas cell of 100 m optical path length.

In this article, based on the TDLAS-WMS technology, a self-developed 4.58-μm CW-QCL combined with a new 41-m MPGC, self-developed QCL driver circuit, harmonic signal demodulation circuit, and Cortex-A5-based human–computer interaction operating system integrated into a field-deployable sensor for the simultaneous detection of CO and N_2_O. The detection precision of the sensor is improved due to the PSO-KELM algorithm embedded in the sensor. Simultaneous high-precision detection of both gases was demonstrated by adjusting the QCL temperature and injection current to cover the two strong absorption lines of CO and N_2_O (CO: 2,179.8 cm^−1^ and N_2_O: 2,179.2 cm^−1^). The use of a single laser for simultaneous detection of two target gases can effectively reduce sensor cost, and the results can be used to analyze the effects of both components on changes in the atmospheric environment.

## Sensor Detection Theory

### WMS Principle

TDLAS technology is now widely used in the detection of trace gases in the atmosphere, which mainly includes direct absorption spectroscopy and modulation spectroscopy techniques (Pi et al., 2019). The modulation spectroscopy techniques include WMS and frequency modulation spectroscopy (FMS). In order to suppress the background noise, WMS technology is usually used, which superimposes a high-frequency sine wave signal on the low-frequency scan signal to modulate the laser wavelength, thus improving the signal-to-noise ratio of the system (Chao et al., 2012).

The transmission of laser light through an absorbing uniform gaseous substance with an optical path length of L can be expressed by the Lambert–Beer law as follows:

$$\frac{I_{\bar{v}}}{I_{0}}=\exp\left(-\alpha \left(\bar{v}\right)\mathrm{LC}\right)=\exp\left(-A\left(\bar{v}\right)\right),$$ (1)

where $I_{\bar{v}}$ and $I_{0}$ is the transmitted and incident laser light intensity at the frequency of $\bar{v}$ , respectively, $\alpha \left(\bar{v}\right)$ is the spectral absorption coefficient, $A\left(\bar{v}\right)$ is the absorbance, and C is the concentration of the measured gas.

The laser frequency is modulated by a high-frequency sine wave signal-modulated scanning signal with an angular frequency of ω . The instantaneous laser frequency is:

$$I\left(t\right)=\bar{v}\left(t\right)+\beta \cos\left(\omega t\right),$$ (2)

where $\bar{v}\left(t\right)$ is the frequency of the scanned signal and *β* is the amplitude of the frequency change of the sinusoidal modulation, which we define as the modulation factor as the ratio of its half-height width Δv to that of the absorption peak (Zheng et al., 2019; Pi et al., 2022):

$$m=\frac{\beta }{\Delta v}.$$ (3)

The Fourier second harmonic (2f) component can be expressed as:

$$H_{2}\left(\bar{v}\right)=\frac{2I_{0}LC}{\pi }\int \left[-\alpha \left(\bar{v}\left(t\right)+\beta \cos\left(\omega t\right)\right)\right]\cos\left(2\omega t\right)d\omega t.$$ (4)

From Eq. 4, the 2f signal is proportional to the gas concentration value, the initial laser light intensity, and the optical path length, etc. By expanding the spectral absorption coefficient at the frequency in a Taylor series, the 2f coefficient can be expressed as:

$$\{\begin{array}{c} I_{2f}=-k\alpha_{0}LCI_{0},\\ k=\frac{2\left[2+m^{2}-2{\left(1+m^{2}\right)}^{\mathrm{0.5}}\right]}{m^{2}{\left(1+m^{2}\right)}^{\mathrm{0.5}}}, \end{array}$$ (5)

where $\alpha_{0}$ is the absorption cross section of the pure gas at the center of the absorption line, and where the modulation factor and the linearity of gas absorption are determined, $I_{2f}\infty LCI_{0}$ , so with L and $I_{0}$ known, the gas concentration can be inverted by the 2f signal (Kluczynski and Axner., 1999; Rui et al., 2017; Lan et al., 2020).

### Algorithm Principle and Application

The extreme learning machine (ELM) has the advantages of simple network structure, fast learning speed, and strong generalization ability, which can overcome the problems of low learning efficiency and tedious parameter setting of traditional neural network algorithms. In recent years, it has started to be applied a lot in the field of soft measurement.

For the algorithm sample $\left(X_{i},t_{i}\right)$ , $X_{i}$ is the collected 2f signal and $t_{i}$ is the corresponding actual gas concentration. The ELM model containing L hidden layer neurons with excitation function g(x) can be expressed as:

$$\sum_{i=1}^{L}\beta_{i}g\left(W_{i}\cdot X_{j}+b_{i}\right)=t_{j},j=1,\cdots ,\mathrm{N,}$$ (6)

where $W_{i}$ is the input weight, $b_{i}$ is the threshold value of the hidden layer neuron (both are randomly generated), and $\beta_{i}$ is the output weight. Equation 6 is expressed in the matrix as:

$$\beta =H^{+}T=H^{+}{\left(HH^{T}\right)}^{-1}\mathrm{T,}$$ (7)

where *H* is the output of the hidden layer neuron, *T* is the target output concentration, and *H* ^*+*^ is the Moore–Penrose generalized inverse of the matrix *H*.

The optimization objective of the ELM is to minimize the training error and the parametrization of the output weights with the following optimization function:

$$\mathrm{minL}=\frac{1}{2}\left\|\beta^{2}\right\|+\frac{1}{2}\gamma \overset{l}{\underset{i=1}{\sum }}\xi_{i}^{2},$$ (8)

$$s.t.h_{i}\left(x\right)\beta =t_{i}-\xi_{i},i=\mathrm{1,2},\cdots ,\mathrm{l,}$$ (9)

where $\xi_{i}$ is the training error and γ is the error penalty factor, which is used to weigh the training error and output weight of the model.

From the Kuhn–Tucker conditions, the final ELM model output weight is obtained:

$$\beta =H^{T}{\left(\frac{1}{\gamma }+HH^{T}\right)}^{-1}T.$$ (10)

In the kernel extreme learning machine (KELM) model, the kernel matrix Ω replaces the random matrix *HH* ^*T*^ in the ELM model, and the kernel matrix can be expressed as:

$$\Omega_{i,j}=HH^{T}=K\left(x_{i},x_{j}\right)=\exp\left(-\frac{x_{i}-x_{j}}{2\sigma^{2}}\right),$$ (11)

where $K\left(x_{i},x_{j}\right)$ is the *i*th row and *j*th column spectral point of ${\text{Ω}}_{i,j}$ . The radial basic function (RBF) is chosen as the kernel function where σ is the kernel function width.

Then, the output concentration expression of the KELM prediction is:

$$f\left(x\right)=h\left(x\right)\beta =\left[\begin{array}{c} K\left(x,x_{1}\right)\\ \vdots \\ K\left(x,x_{l}\right) \end{array}\right]{\left(\frac{1}{\gamma }+\Omega \right)}^{-1}T.$$ (12)

The error penalty factor γ and the kernel parameter $\sigma^{2}$ are the two key parameters for KELM optimization. In this article, the particle swarm optimization (PSO) algorithm is used to optimize γ and $\sigma^{2}$ (Lu et al., 2017; Fu et al., 2019; Afzal et al., 2021).

## Sensor Structure and Configuration

### Absorption Line Selection

**FIGURE 1:**
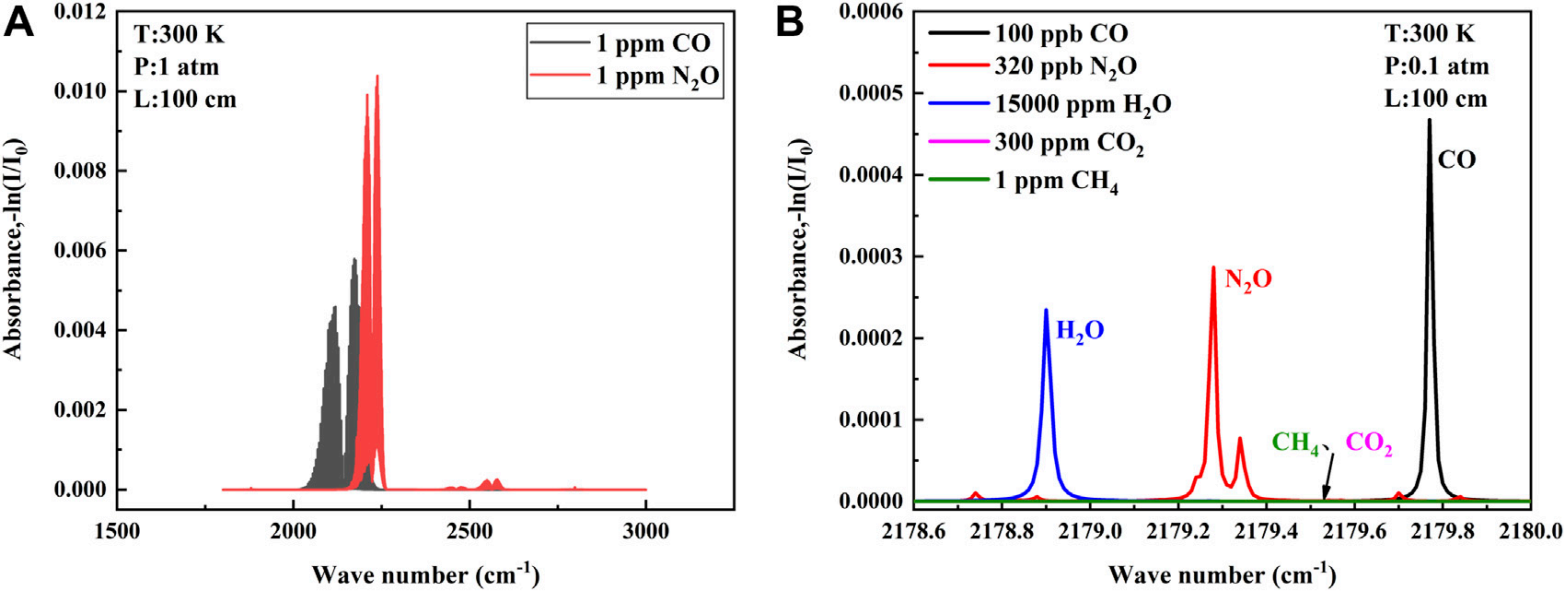
**(A)** Absorption bands of CO and N2O provided by the database in the range of 1800-3000 cm^−1^. **(B)** Absorption lines of selected CO and N_2_O and other interfering gases at low pressure.

There are higher absorption line strength of CO and N_2_O in the mid-infrared waveband than that in the near-infrared waveband, so the mid-infrared waveband was chosen for detection. The absorption lines of CO gas molecules and N_2_O gas molecules at wave numbers of 1,800–3,000 cm^−1^ at a temperature of 25°C are shown in Figure 1A (data from the HITRAN database). As can be seen from the figure, each of the two gases has a strong absorption band and a weak absorption band. For single gas detection, the absorption line of the strong absorption band is chosen as much as possible, but for simultaneous detection of both gases, the overlapping part of the absorption bands of the two gases needs to be considered. Only when the appropriate absorption lines are selected can the QCL cover both gas absorption lines in a single current scan, thus enabling simultaneous detection and greatly reducing the cost of the detection system (Guo et al., 2020).

To find absorption lines in the overlapping absorption bands of the two gases, we must first ensure that the absorption line strength of the two gases is sufficient to ensure the signal amplitude and signal-to-noise ratio of our back-end. Second, the interference of other gases should be avoided. The atmospheric environment is complex with more interfering gases, and the gases with more content in the atmosphere need to be considered, generally including H_2_O, CO_2_, and CH_4_, etc. Finally, the distance and line shape of the absorption lines of the two gases need to be considered. If the two lines are too far apart, it is difficult to be covered within a single current scan of the QCL, and if they are too close together, it is easy to generate mutual interference, which affects the detection precision. Air pressure usually is reduced to separate two similar absorption lines, but the sensitivity is also affected when the pressure is reduced. All aspects need to be considered (Yu et al., 2016; Song et al., 2018). The final absorption lines selected according to the aforementioned point rules are shown in Figure 1B, where the CO absorption line is located at 2,179.77 cm^−1^ and the N_2_O absorption line is located at 2,179.28 cm^−1^. Each gas is proportioned according to the atmospheric environment, and the absorption lines can be separated without cross influence at 0.1 atm. The QCL with narrow line width can cover two target absorption lines in a single current scan without introducing interference from H_2_O, meeting the requirement of simultaneous detection of two gases in the atmospheric environment.

### CO and N_2_O Sensor Overview

**FIGURE 2:**
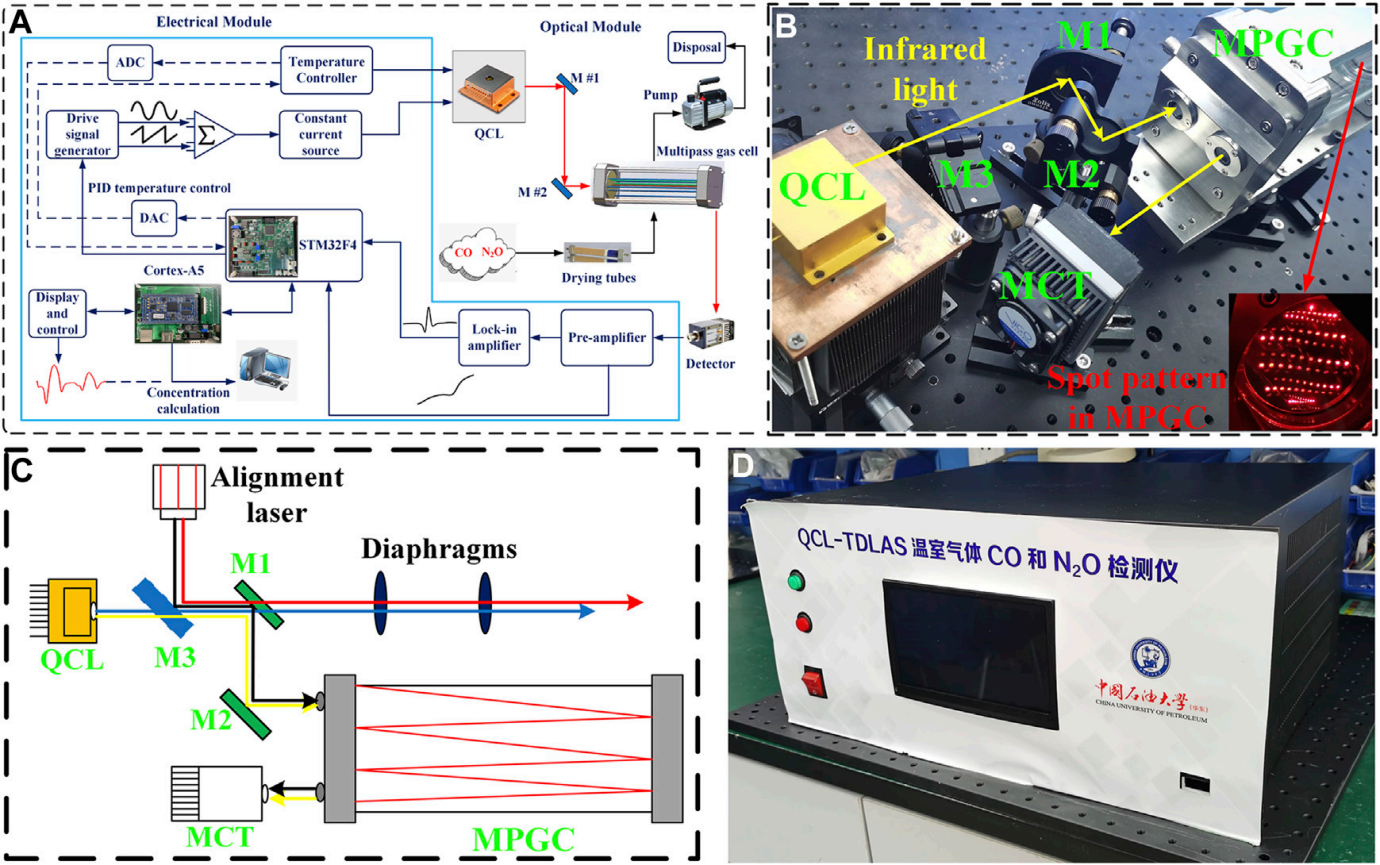
**(A)** Block diagram of CO and N_2_O sensor system, which mainly consists of an optical module and an electrical module. **(B)** Photograph of CO and N_2_O sensor optical module. **(C)** Schematic of the beam tracing using alignment laser. **(D)** Photograph of CO and N_2_O sensor electrical module.

The functional block diagram of the designed CO and N_2_O sensor is shown in Figure 2A, which mainly includes optical and electrical parts. A DB15 cable is used to connect these two parts to drive the QCL and to provide power to the optical part. The optical part: a compact optical structure was designed for coupling the mid-infrared free-space-emitting laser beam into the gas cell and delivering the output to the detector, as exhibited in Figure 2B. The 4.58-μm QCL developed independently by the Institute of Semiconductor Research, Chinese Academy of Sciences, was selected. With the tuning range of 2,178 to 2,181.7 cm^−1^ at a laser temperature of 30°C, the two target gas absorption lines can be scanned simultaneously, and the QCL output can reach a peak power of 25 mW at an injection current of 400 mA. An MPGC with a physical size of 27 × 8.4 × 12 cm^3^ provides a sealed environment for the interaction between the infrared light and the target gas and offers a 41 m effective optical path length after 220 reflections. The laser beam is emitted from the emitter and received by the detector. A mercury–cadmium–telluride (MCT) detector (VIGO System, Model PVI-4TE-8) was applied to convert the absorbed infrared light to an electrical signal, which has a detection rate of $D^{*}=2.9\times 10^{10}cm\sqrt{Hz}/W$ .

A visible laser and a flip mirror were applied for beam tracing, which is described in Figure 2C. There are four alignment steps, in which the beam paths are represented by lines in blue (step 1), red (step 2), black (step 3), and yellow (step 4), respectively. Step 1: first turn on the QCL and make the infrared light through the center of both diaphragms. Step 2: then, guide the red alignment beam also through the center of both diaphragms with the help of a flip mirror (M3). This means that the infrared beam is co-linear with the red alignment beam. Step 3: the two plane mirrors (M1 and M2) were placed to reflect the red alignment beam into the MPGC and obtained a specific spot pattern shown by the inset in Figure 2B, which means that the optimum incident angle was found. The visible beam is also used to fix the MCT position. Step 4: finally, the flip mirror was switched off to make the infrared light being reflected into the MPGC and reach the MCT. Therefore, the invisible infrared light was successfully traced by the visible beam from the alignment laser (Liu et al., 2019).

The electrical part: the photograph of the electrical module is shown in Figure 2D. The STM32F4 from STMicroeletronics (STM) was chosen as the core micro-control unit (MCU) for the electrical part. The STM32F4 microcontroller-based driver and demodulation circuit were designed to form the QCL driving signal by superimposing a 7.8 kHz sine wave signal on a 5 Hz sawtooth wave scan signal. The voltage signal is converted into a current signal through a constant current source module to drive the QCL. The voltage signal output from the MCT is demodulated by the preamplifier and lock-in amplifier to produce the 2f signal which responds to the gas concentration. A WAVELENGTH’s PTC-10 thermostat is selected for temperature control with a maximum thermoelectric cooler (TEC) current of ±10 A and temperature stability of 0.0012°C. Temperature setting and adjustment by STM32 were performed, combined with proportional-integral-derivative (PID) algorithm to keep the target temperature within an acceptable range with the real-time temperature. At the same time, a wavelength drift compensation algorithm is applied to correct wavelength drift due to temperature changes. This algorithm compensates for wavelength changes caused by temperature fluctuations by varying the laser operating current in real time, so that the wavelength always covers the two gas absorption peaks. The ARM Cortex-A5 processor combined with a capacitive touch screen is applied for the QCL parameter setting and absorption line display, and the concentration prediction is performed in real time by PSO-KELM algorithm embedding.

### Driver and Demodulation Circuit

**FIGURE 3:**
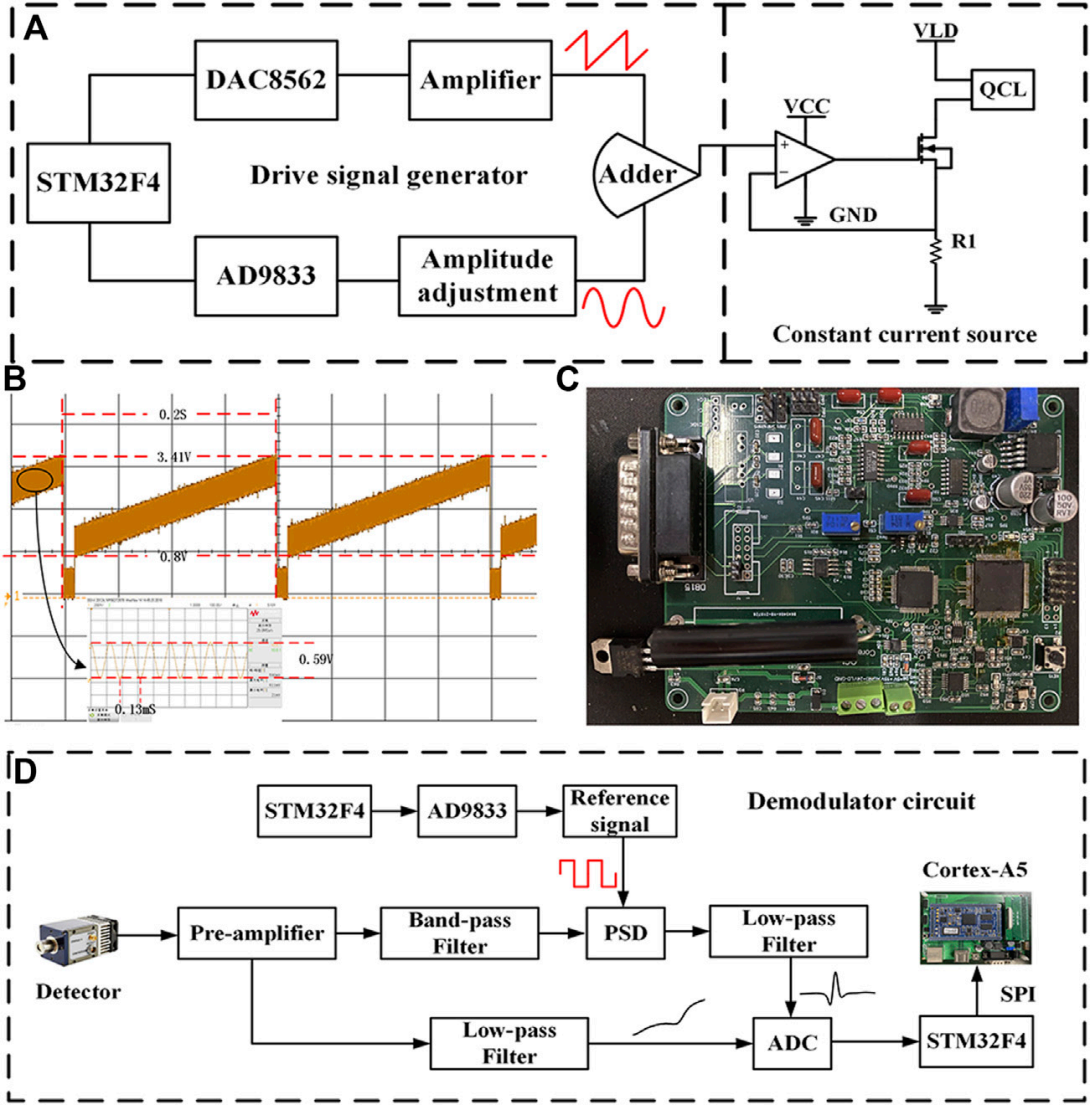
**(A)** Block diagram of QCL driver circuit. **(B)** Voltage driver signal waveform. **(C)** Photograph of the self-developed board-level QCL driver and demodulation circuit. **(D)** Block diagram of demodulation circuit.

In order to meet the demand for CO and N_2_O sensors, the driver and demodulation circuit of the board-level QCL was developed independently. The block diagram of the QCL driver circuit is shown in Figure 3A. The STM32 controls the DAC8562 chip and the AD9833 chip to generate a low-frequency sawtooth signal at 5 Hz and a sine wave signal at 7.8 kHz, respectively. The aforementioned two signals are superimposed by an additive circuit to generate the voltage driver signal for the QCL. The constant current source circuit converts it into a current driver signal, which is injected into the QCL to modulate the QCL output wavelength, thus covering the absorption lines of both CO and N_2_O gases simultaneously. In the constant current source circuit, the negative feedback principle of the operational amplifier and the characteristics of the MOSFET tube make the output current dynamically stable, where R1 is the sampling resistor, the precision of which affects the stability of the output current, and the high precision sampling resistor is selected for the large driver current of QCL. Stable constant-current source circuitry stabilizes the output wavelength of the QCL, thereby avoiding the effects of spectral drift and improving sensor precision. The waveform of the voltage driver signal is shown in Figure 3B, and the photograph of the developed QCL driver and demodulation circuit is shown in Figure 3C.

The block diagram of the harmonic signal demodulation circuit is shown in Figure 3D, the core part of which is the lock-in amplifier. First, the output signal of the MCT is amplified by the preamplifier circuit and then divided into two ways, one way to get the optical power signal after low-pass amplification; the other way is filtered by the band-pass filter circuit with the center frequency of 15.6 kHz to remove part of the interference and then enters the lock-in amplifier with the two-fold square wave reference signal for phase sensitive detection (PSD) (Chen et al., 2017). The 2f signal from the lock-in amplifier and the optical power signal from the other side are collected using the analog-to-digital converter (ADC) and passed to the Cortex-A5 processor for normalization via serial peripheral interface (SPI) communication.

## Sensor Performance Experiments

### Mixing and Proportioning Experiments

**FIGURE 4:**
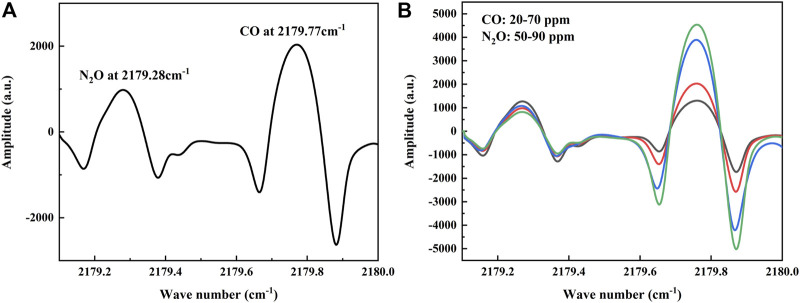
**(A)** Absorption lines of CO and N_2_O at different concentrations detected by the sensor, in agreement with the information given in the database. **(B)** Absorption lines of mixed ratios of CO and N_2_O detected by the sensor.

To verify the performance of the sensor, a series of experiments were conducted in the laboratory. In the experiment, a vacuum pump was used to draw the gas out of the MPGC to create a low-pressure detection environment that facilitates the separation of the two absorption lines. The MPGC pressure is maintained at 0.1 atm using a pressure controller (TESCOM, Model ER-5000SI-1), and the MPGC inlet flow rate is controlled at 1 slm (standard liters/min). At the same time, a Panasonic DP-101 pressure sensor is used to display the pressure of the MPGC in real time. Therefore, 30 ppm of CO and 70 ppm of N_2_O were introduced at 25°C, and the absorption lines (which is the 2f signal obtained from the demodulation circuit) of the two gases were detected by the QCL in a single current scan as shown in Figure 4A. It is consistent with the database, and at this time the two gas absorption lines are far away from each other without cross-over effects.

In order to verify the effect of dynamic changes of the two gases on each other’s absorption line, experiments with random changes in the concentrations of CO and N_2_O were conducted under the above low-pressure conditions (Pi et al., 2021). A total of five groups of high concentration gas mixture ratios were conducted, where CO varied from 20–70 ppm and N_2_O varied from 50–90 ppm, and the collected 2f signals are shown in Figure 4B. A randomly varying background gas has less effect than the other gas, so that both gases can be detected simultaneously.

### Algorithm Comparison

**FIGURE 5:**
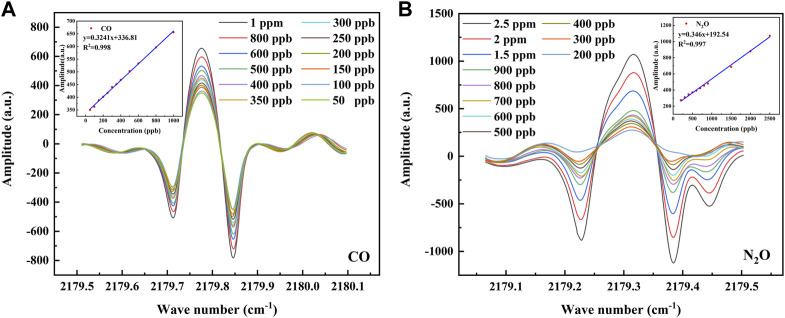
**(A)** The 2f signal at CO concentrations of 50–1000 ppb and the linear relationship between the set concentrations of CO and its 2f peak-to-peak value. **(B)** The 2f signal at N_2_O concentrations of 200–2500 ppb and the linear relationship between the set concentrations of N_2_O and its 2f peak-to-peak value.

Before performing the concentration calibration, it is first necessary to verify the linearity between the set concentration and the 2f signal detected by the sensor, and for this purpose, a concentration step experiment is performed for CO and N_2_O. The CO was rationed in a concentration gradient and divided into 12 groups of 50, 100, 150, 200, 250, 300, 350, 400, 500, 600, 800, and 1,000 ppb; N_2_O was rationed in a concentration gradient and divided into 11 groups of 200, 300, 400, 500, 600, 700, 800, 900, 1,500, 2000, and 2,500 ppb. The 2f signal output from the demodulation circuit inside the sensor is first processed by averaging 10 times and Savitzky–Golay (S-G) filtering to eliminate part of the noise. Then, the optical power signal is divided by the raw 2f signal to eliminate the intensity modulation of the optical power, which is the normalization process. The processed 2f signals and the linear fits of their peak-to-peak values to the set concentrations are shown in Figure 5, where the linear correlation is 0.998 for CO and 0.997 for N_2_O. This indicates that the peak-to-peak value of the 2f signal detected by the sensor at lower concentrations has a good linear relationship with the set concentration, and the peak-to-peak value of this 2f signal can be used to directly predict the concentrations of CO and N_2_O.

To verify the performance of the PSO-KELM algorithm, the results were compared with the commonly used BP neural network algorithm and PLS algorithm using the detected data of N_2_O. A total of 140 N_2_O absorption lines in 10 groups were used for the five-fold calibration method, of which 112 absorption lines were taken as the training set and 28 absorption lines as the test set. The PSO algorithm is used to optimize the kernel radius $\sigma^{2}$ and the penalty factor γ of the RBF kernel function. It was found that the highest prediction precision was achieved for the test set of 28 samples when $\gamma =1.595\times 10^{4}$ and $\sigma^{2}=2.537\times 10^{10}$ .

**FIGURE 6:**
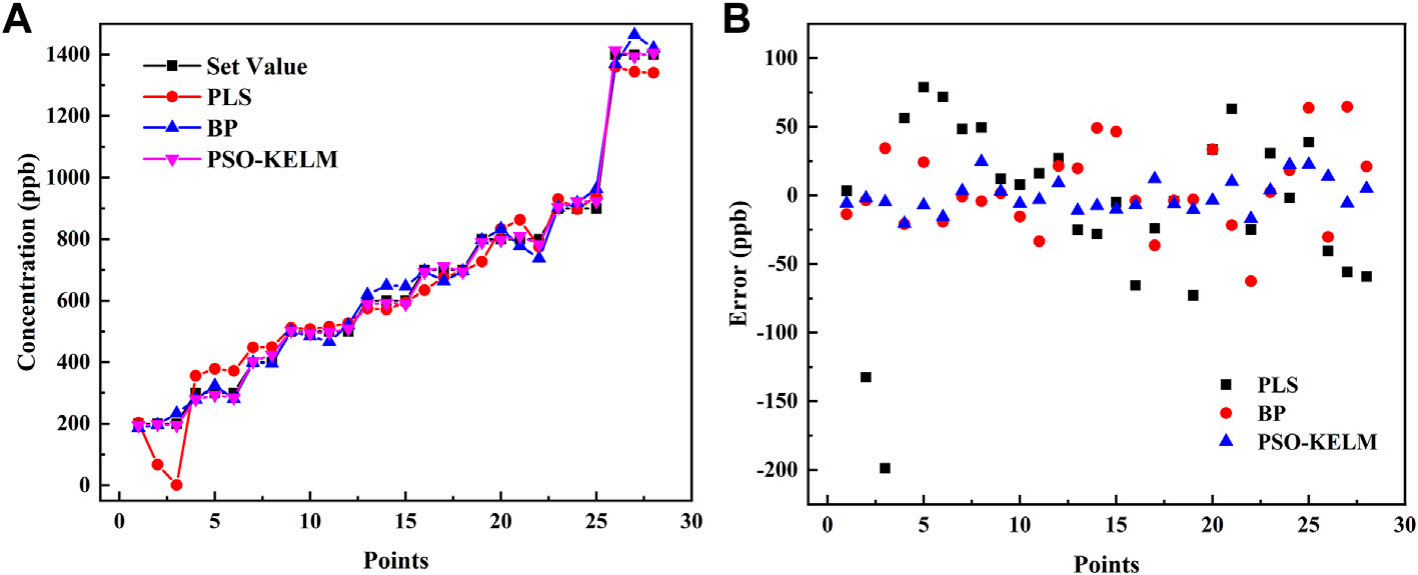
**(A)** Comparison of the set values and predicted results of the BP neural network, PLS and PSO-KELM algorithms. **(B)** Error comparison of the set values and predicted results of the BP neural network, PLS and PSO-KELM algorithms.

The concentration prediction results of the PSO-KELM algorithm were compared with PLS and BP neural networks (with ReLU as the activation function), and the results are shown in Table 1. The PSO-KELM prediction results had the lowest RMSE and the closest linear correlation coefficient to 1. Its RMSE is 3.14 times lower than that of BP neural network and 5.14 times lower than that of PLS. Therefore PSO-KELM has the highest prediction precision, and this algorithm was chosen to perform CO and N_2_O concentration prediction at the sensor. The fitting results of the three algorithm models of BP neural network, PLS, and PSO-KELM are shown in Figure 6.

**TABLE 1:** Comparison of fitting precision of BP neural network, PLS, and PSO-KELM algorithms for the step test of N_2_O gas.

Algorithm	RMSE/ppb	*R* ^2^
PSO-KELM	11.688	0.9997
BP neural network	36.757	0.9975
PLS	60.078	0.9683

### Group Interference Experiments

**FIGURE 7:**
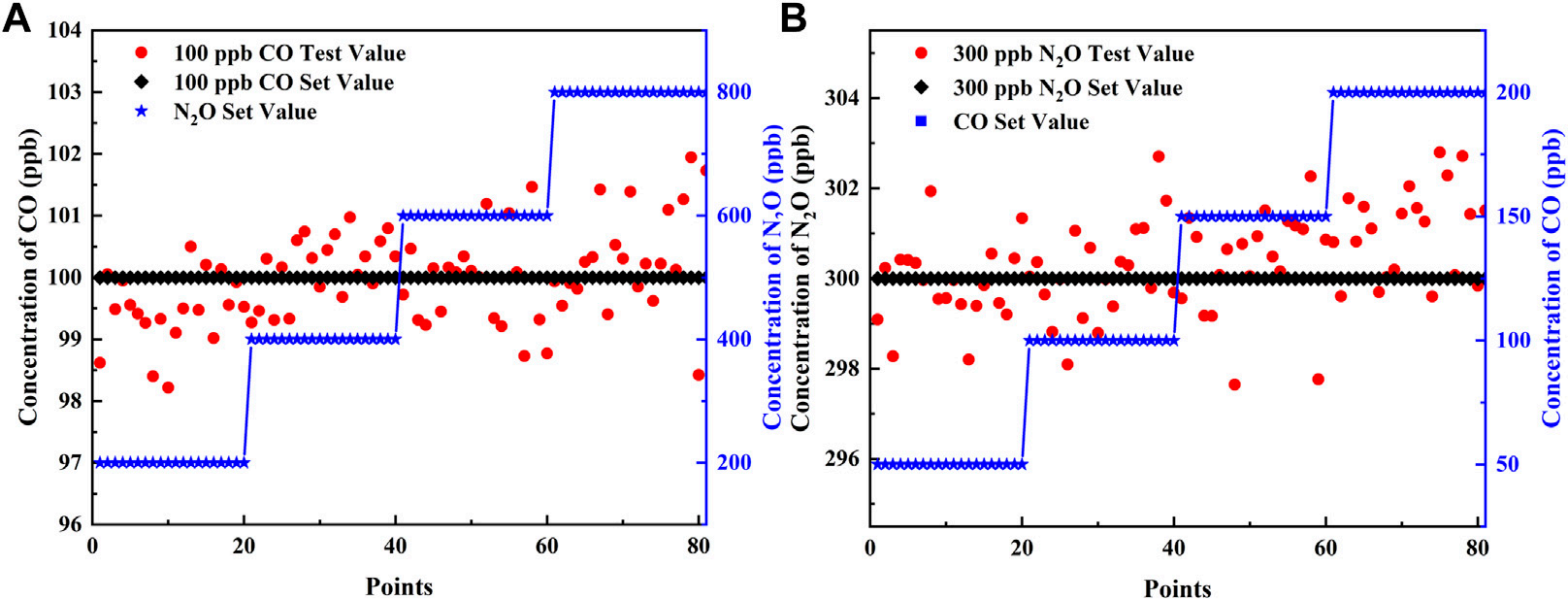
**(A)** Different concentrations of N_2_O interfere with 100 ppb of CO. **(B)** Different concentrations of CO interfere with 300 ppb of N_2_O.

To verify the precision of the simultaneous detection of the two gases and the robustness of the sensor, component interference experiments were performed for fixed concentrations of CO and N_2_O, respectively. To simulate the content of the two gases in the air, the experiment was divided into two groups: one group fixed the concentration of CO at 100 ppb and N_2_O in steps of 200, 400, 600, and 800 ppb; the other group fixed the concentration of N_2_O at 300 ppb and CO in steps of 50, 100, 150, and 200 ppb. The aforementioned PSO-KELM algorithm was applied for gas concentration prediction, when the concentration of one of the gases varies according to a gradient, the concentration stability of the other gas is shown in Figure 7. When 100 ppb of CO was disturbed by different concentrations of N_2_O, the predicted values of CO fluctuated between 98.2 and 101.9 ppb with an error of 0.03 ppb; when 300 ppb of N_2_O was disturbed by different concentrations of CO, the predicted values fluctuated between 297.6 and 302.8 ppb with an error of 0.36 ppb. Therefore, the sensor has good robustness, and the error meets the precision requirement.

### Long-Term Stability Experiments

**FIGURE 8:**
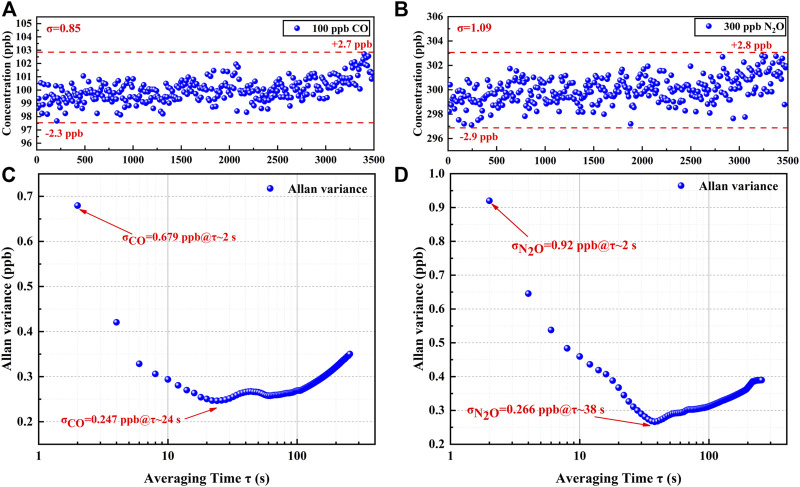
**(A)** 60 mins measurement results of CO at constant concentration. **(B)** 60 mins measurement results of N_2_O at constant concentration. **(C)** Allan variance analysis of the sensor based on the data shown in **(A)**. **(D)** Allan variance analysis of the sensor based on the data shown in **(B)**.

To verify the stability of the sensor and the precision of the detection, 100 ppb of CO and 300 ppb of N_2_O were introduced, and the fluctuations were detected over 60 min. CO and N_2_O concentration levels were obtained from the PSO-KELM and plotted in Figures 8A,B. The CO concentration fluctuated between 97.7 and 102.7 ppb with a detection standard deviation of 0.85 ppb, and the N_2_O concentration fluctuated between 297.1 and 302.8 ppb with a detection standard deviation of 1.09 ppb, indicating that the established sensor has the high stability. An Allan deviation analysis was employed on the data to characterize the stability, as plotted on a log–log scale versus the averaging time in Figures 8C,D. The LoD of 0.68 ppb for CO and 0.92 ppb for N_2_O for a 2 s averaging time and the minimum LoD of 0.25 ppb for CO and 0.27 ppb for N_2_O for the optimum averaging time of 24 and 38 s, respectively, were obtained based on the analysis. The stability of the sensor is the best when the averaging time is in the range of 24–38 s. However, in practical applications, the averaging time of the sensor is still set to 2 s in order to ensure a faster response.

### Field Deployment of CO and N_2_O Sensor

**FIGURE 9:**
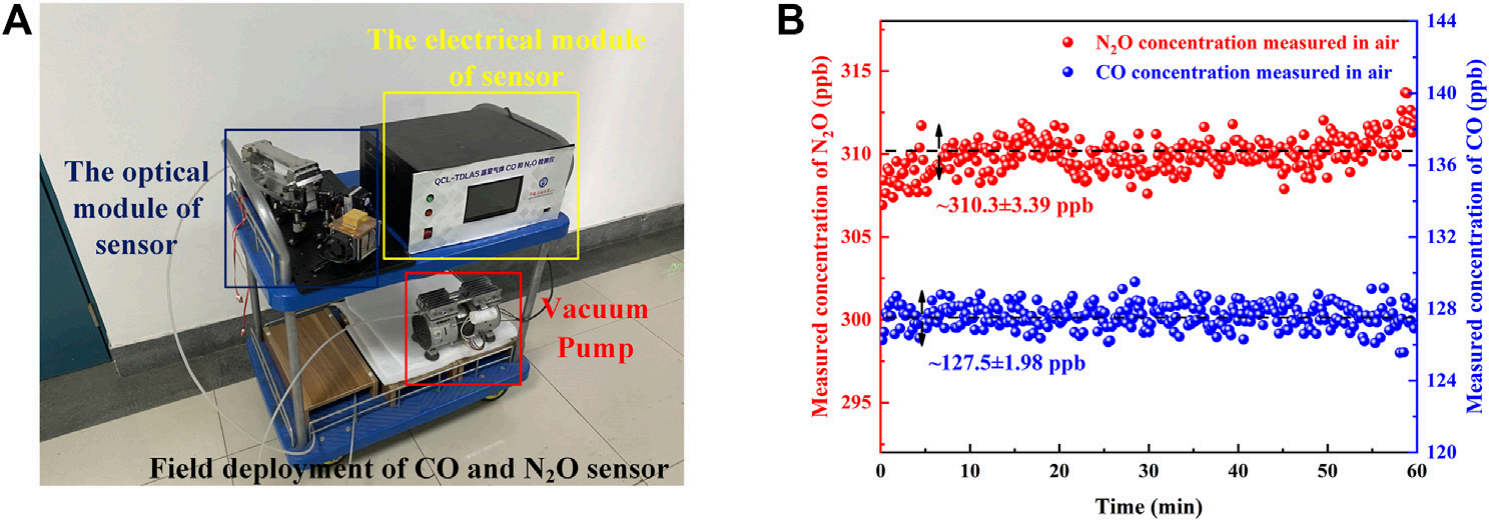
**(A)** Photograph of the sensor for the field deployment. **(B)** The real-time measured concentrations of the sensor.

After verification of the sensor performance and concentration calibration in the laboratory, the sensor was deployed in the field. The sensor was deployed in the Yifu Laboratory Building of the China University of Petroleum (East China). The photograph of the sensor system is shown in Figure 9A, which mainly includes the electrical module, optical module, and vacuum pump. Under the control of the pressure controller, the air is constantly pumped in through the MPGC inlet port and out through the outlet port to maintain the low-pressure conditions required for detection (Zheng et al., 2020). By covering both CO and N_2_O absorption lines, simultaneous detection of CO and N_2_O from the air was performed for ∼1 h. The real-time detected concentrations of the sensor are shown in Figure 9B. According to the detection results, the CO concentration of the air is determined to be ∼127.5 ± 1.98 ppb, and the N_2_O concentration of the air is determined to be ∼310.3 ± 3.39 ppb. Various chemical experiments are conducted in the Yifu Laboratory Building, so the concentration of CO in the air is slightly higher.

## Conclusion

In this article, we reported a compact field deployable sensor for simultaneous detection of CO and N_2_O in the atmosphere in which the QCL with a central wavelength of 4.58 μm and an MPGC with an optical path length of 41 m was applied. Also, the PSO-KELM algorithm is embedded in the sensor as a way to improve the detection precision of the system. Then, the gas detection experiments are conducted to verify the sensor performance. The results show that the sensor has a good linear response for detecting low concentrations of CO and N_2_O, and the PSO-KELM has lower RMSE than the commonly used BP neural network and PLS algorithms, which greatly improves the detection precision of the sensor. The LoD of 0.25 ppb for CO and 0.27 ppb for N_2_O at the averaging time of 24 and 38 s was demonstrated, respectively. The application of self-developed QCL, hardware circuit system, and embedded software system greatly reduces the cost of the sensor and simplifies the sensor structure. Field deployment of the sensor was reported for simultaneous detection of CO and N_2_O in the air, which validated the normal operation of the sensor system. The sensor can be used to detect CO and N_2_O contents in the air in different regions and different environments, which is important for environmental monitoring and research on Earth’s climate change.

## Data Availability

The original contributions presented in the study are included in the article/Supplementary Material; further inquiries can be directed to the corresponding author.
