# Supplementary material for: Performance of a Mid-Infrared Sensor for Simultaneous Trace Detection of Atmospheric CO and N2O Based on PSO-KELM
Source: Front Chem. 2022 Jul 14;10:930766. doi: 10.3389/fchem.2022.930766 (PMC9333160; doi:10.3389/fchem.2022.930766)
Supplement: Supplementary file 1 [file DataSheet1.docx]

Supplementary Material

# Supplementary Figures and Tables

## Supplementary Figures


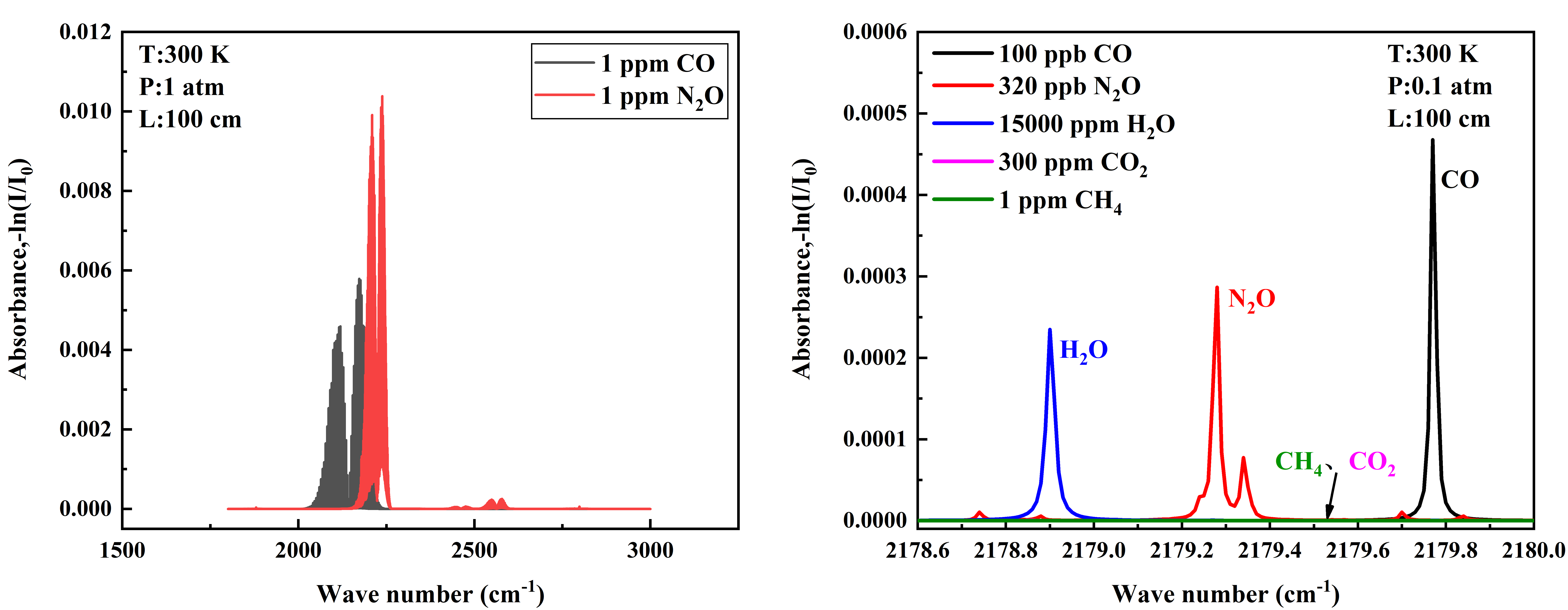


**Supplementary Figure 1.** **(a)** Absorption bands of CO and N_2_O provided by the database in the range of 1800-3000 cm^-1^. **(b)** Absorption lines of selected CO and N_2_O and other interfering gases at low pressure.


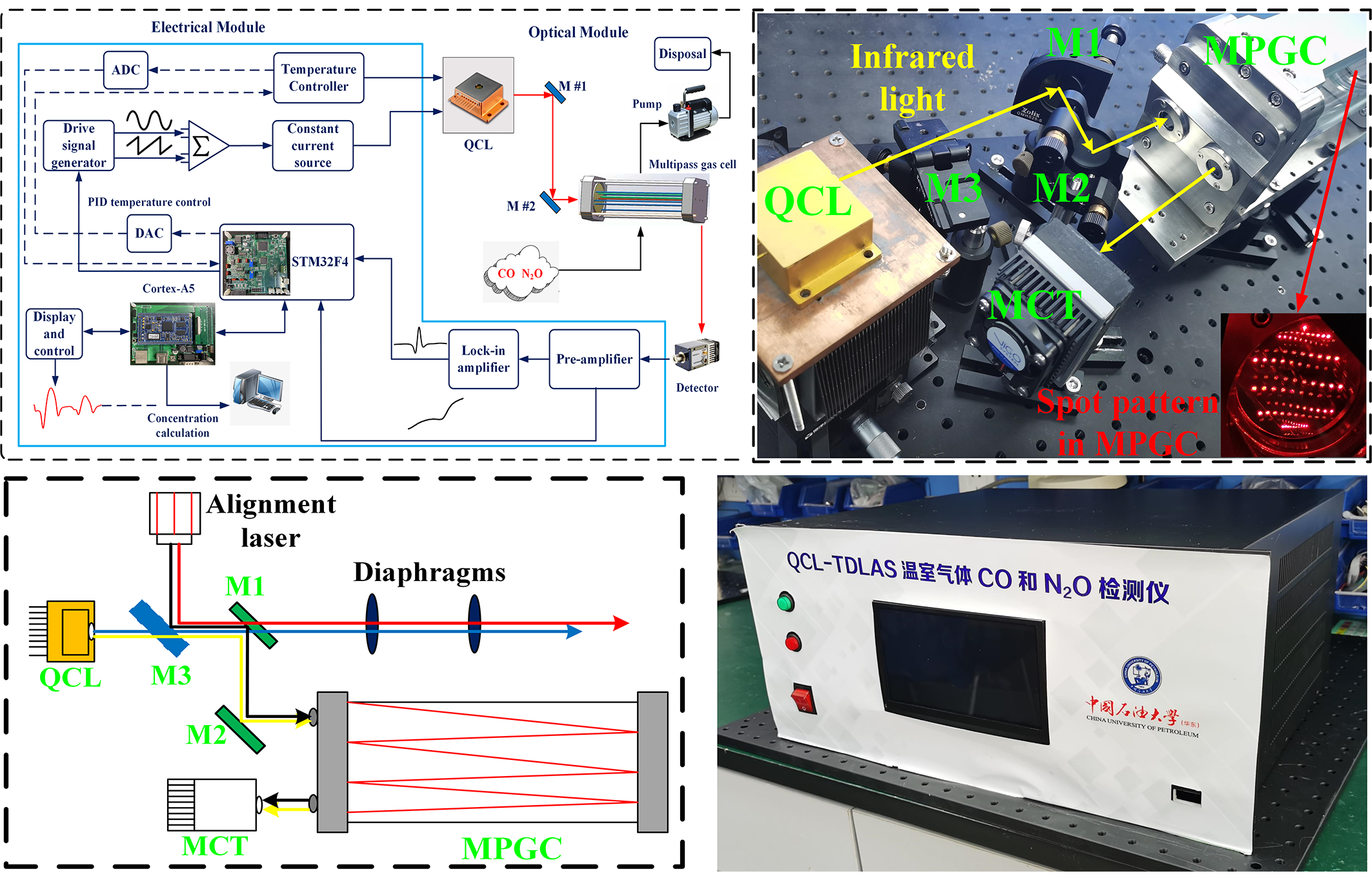


**Supplementary Figure 2.** **(a)** Block diagram of CO and N_2_O sensor system, which mainly consists of an optical module and an electrical module. **(b)** Photograph of CO and N_2_O sensor optical module. **(c)** Schematic of the beam tracing using alignment laser. **(d)** Photograph of CO and N_2_O sensor electrical module.


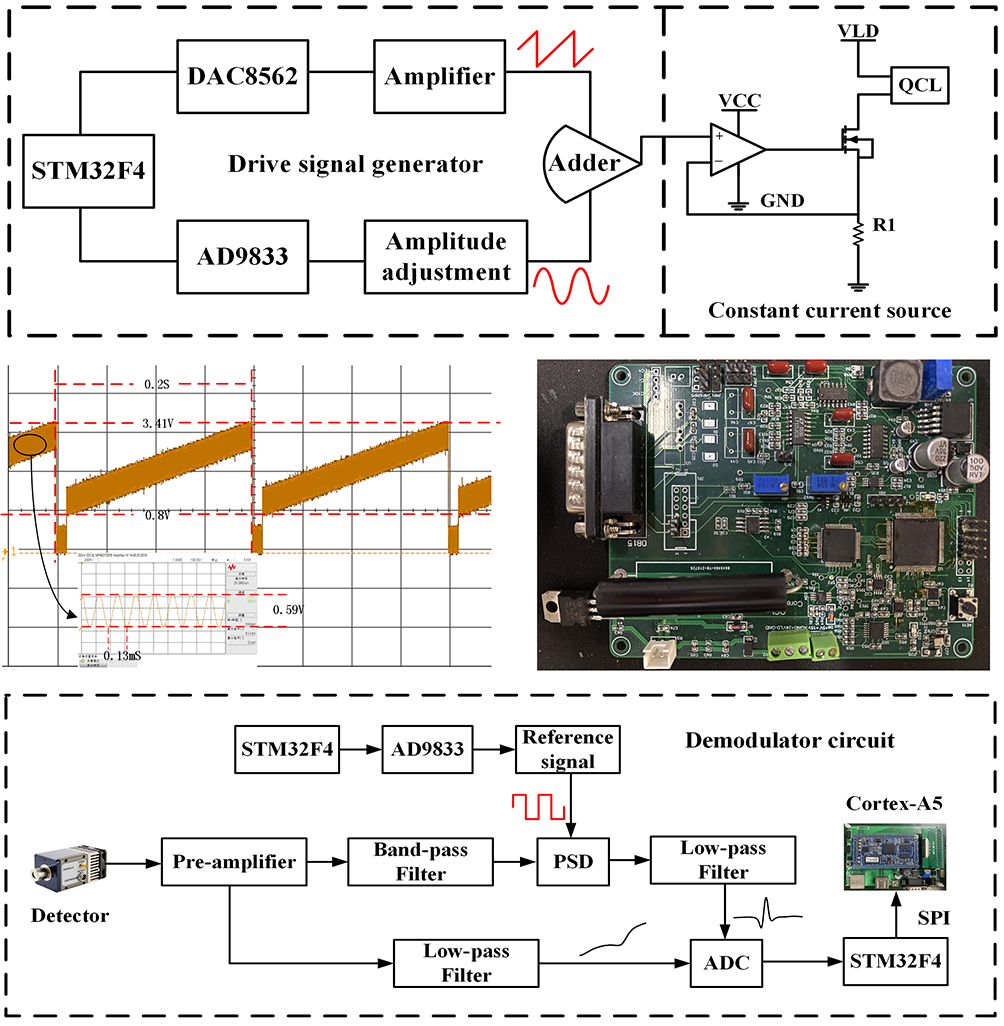


**Supplementary Figure 3.** **(a)** Block diagram of QCL driver circuit. **(b)** Voltage driver signal waveform. **(c)** Photograph of the self-developed board-level QCL driver and demodulation circuit. **(d)** Block diagram of demodulation circuit.


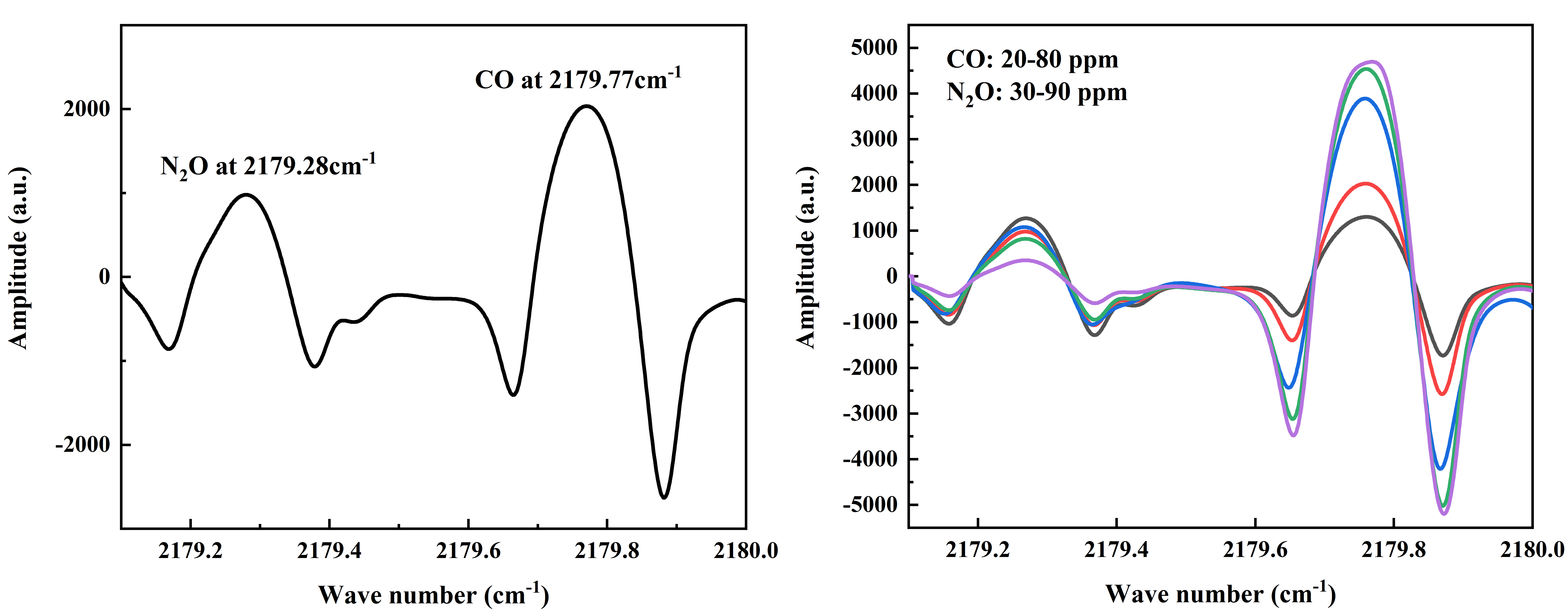


**Supplementary Figure 4.** **(a)** Absorption lines of CO and N_2_O at different concentrations detected by the sensor, in agreement with the information given in the database. **(b)** Absorption lines of mixed ratios of CO and N_2_O detected by the sensor.


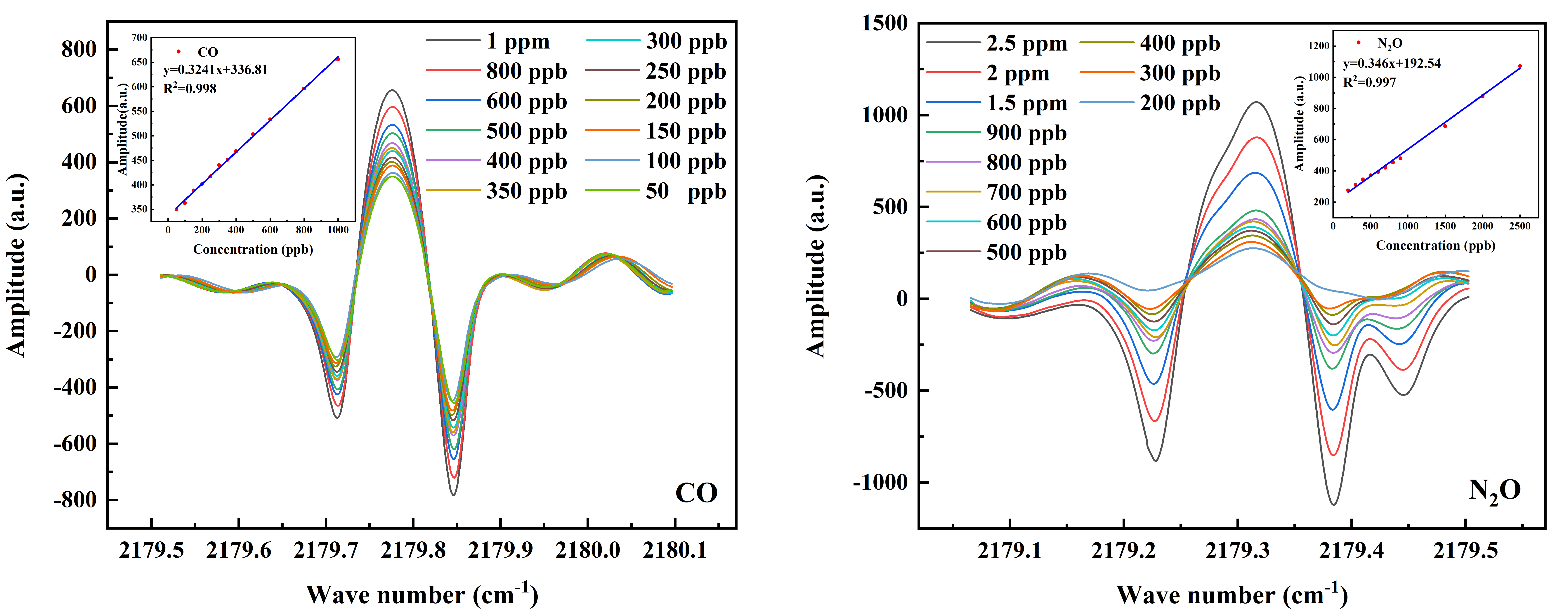


**Supplementary Figure 5.** **(a)** The 2f signal at CO concentrations of 50-1000 ppb and the linear relationship between the set concentrations of CO and its 2f peak-to-peak value. **(b)** The 2f signal at N_2_O concentrations of 200-2500 ppb and the linear relationship between the set concentrations of N_2_O and its 2f peak-to-peak value.


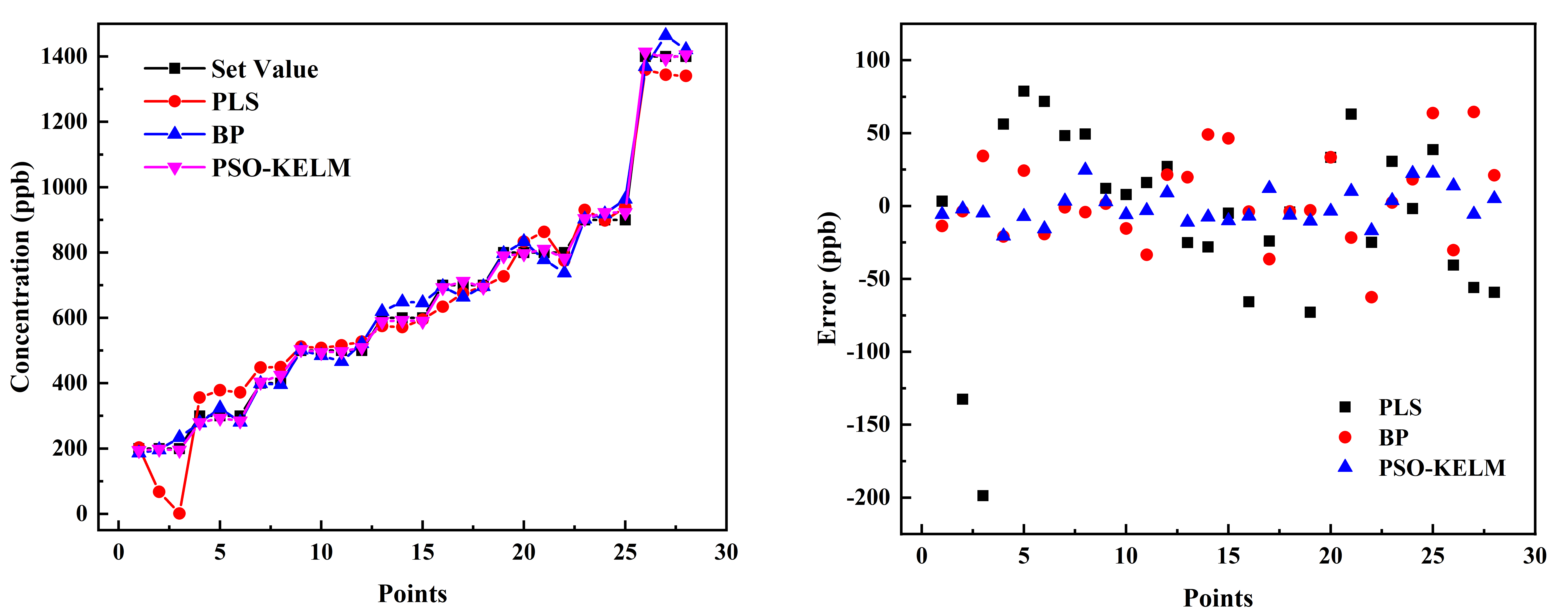


**Supplementary Figure 6.** **(a)** Comparison of the set values and predicted results of the BP neural network, PLS and PSO-KELM algorithms. **(b)** Error comparison of the set values and predicted results of the BP neural network, PLS and PSO-KELM algorithms.


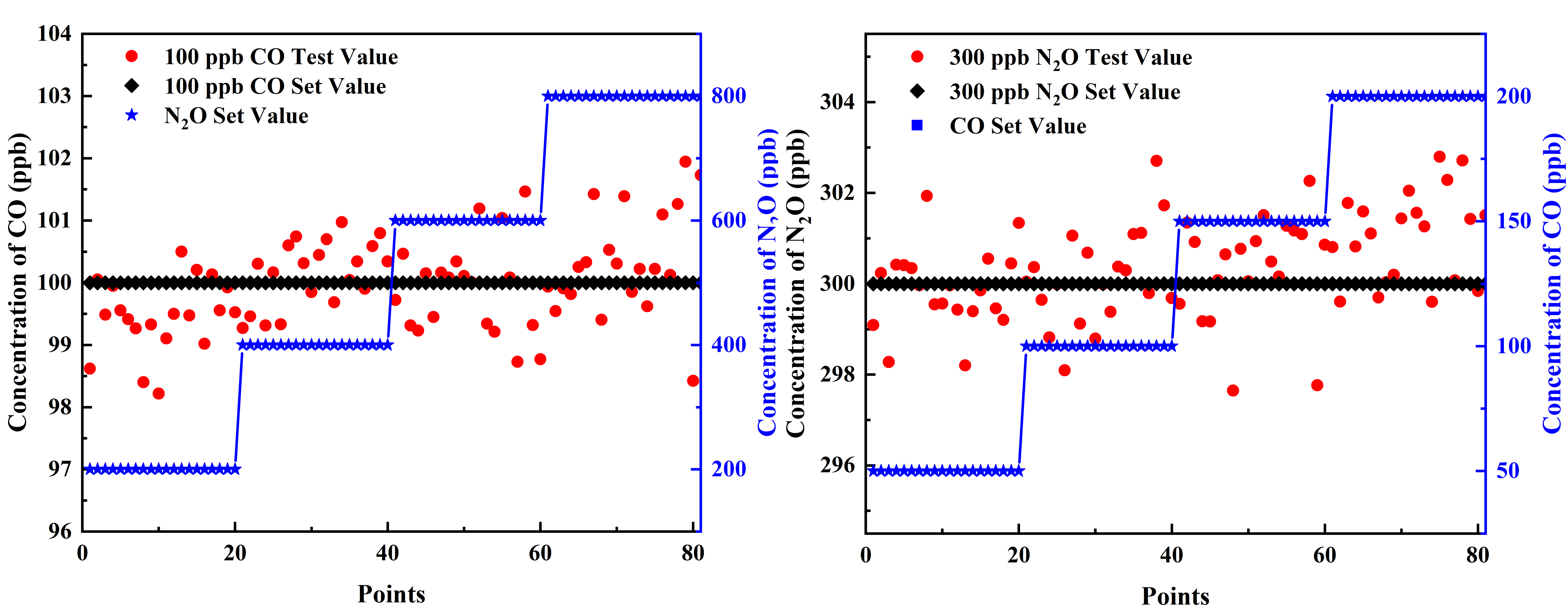


**Supplementary Figure 7.** **(a)** Different concentrations of N_2_O interfere with 100 ppb of CO. **(b)** Different concentrations of CO interfere with 300 ppb of N_2_O.


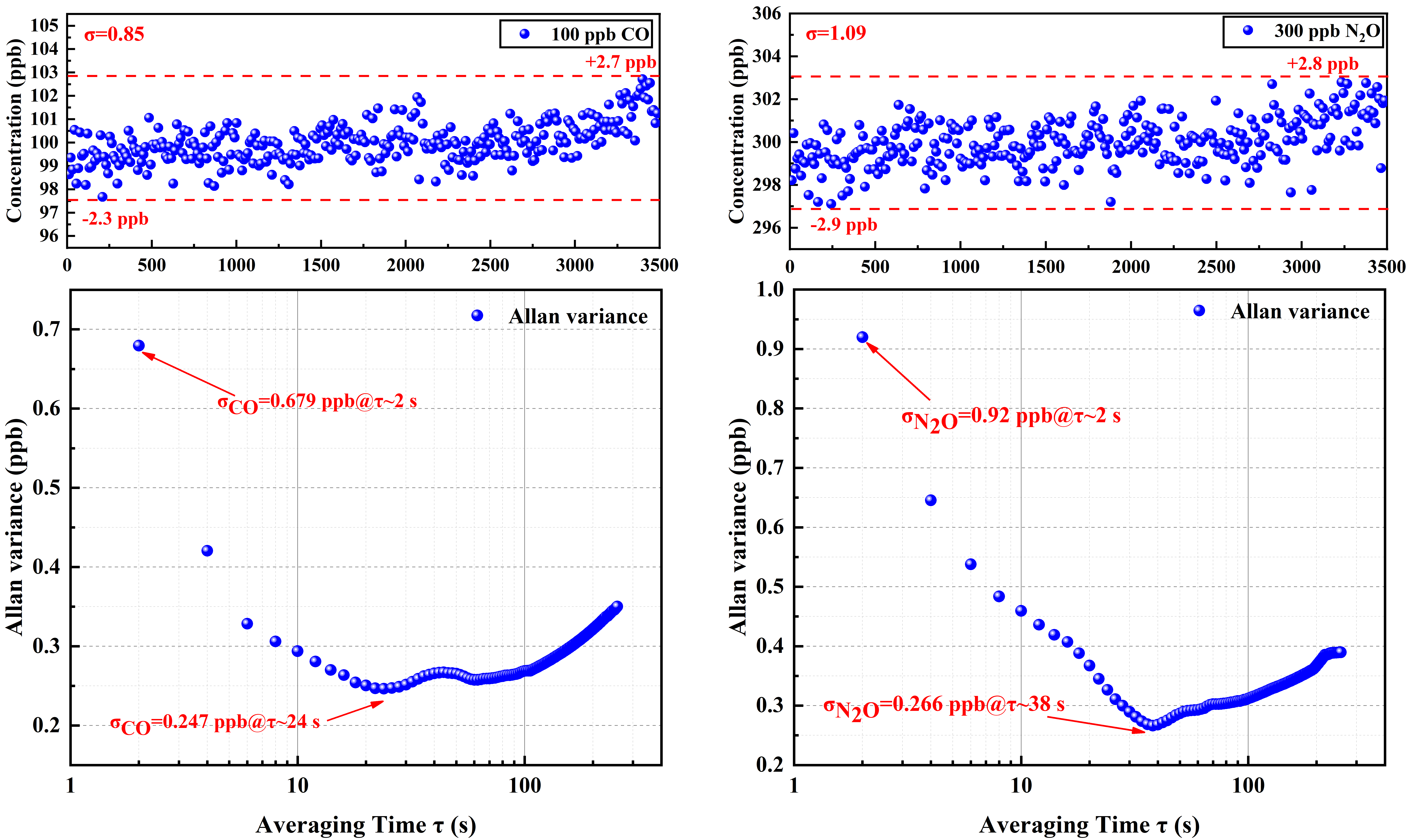


**Supplementary Figure 8.** **(a)** 60 mins measurement results of CO at constant concentration. **(b)** 60 mins measurement results of N_2_O at constant concentration. **(c)** Allan variance analysis of the sensor based on the data shown in Figure.8(a). **(d)** Allan variance analysis of the sensor based on the data shown in Figure.8(b).


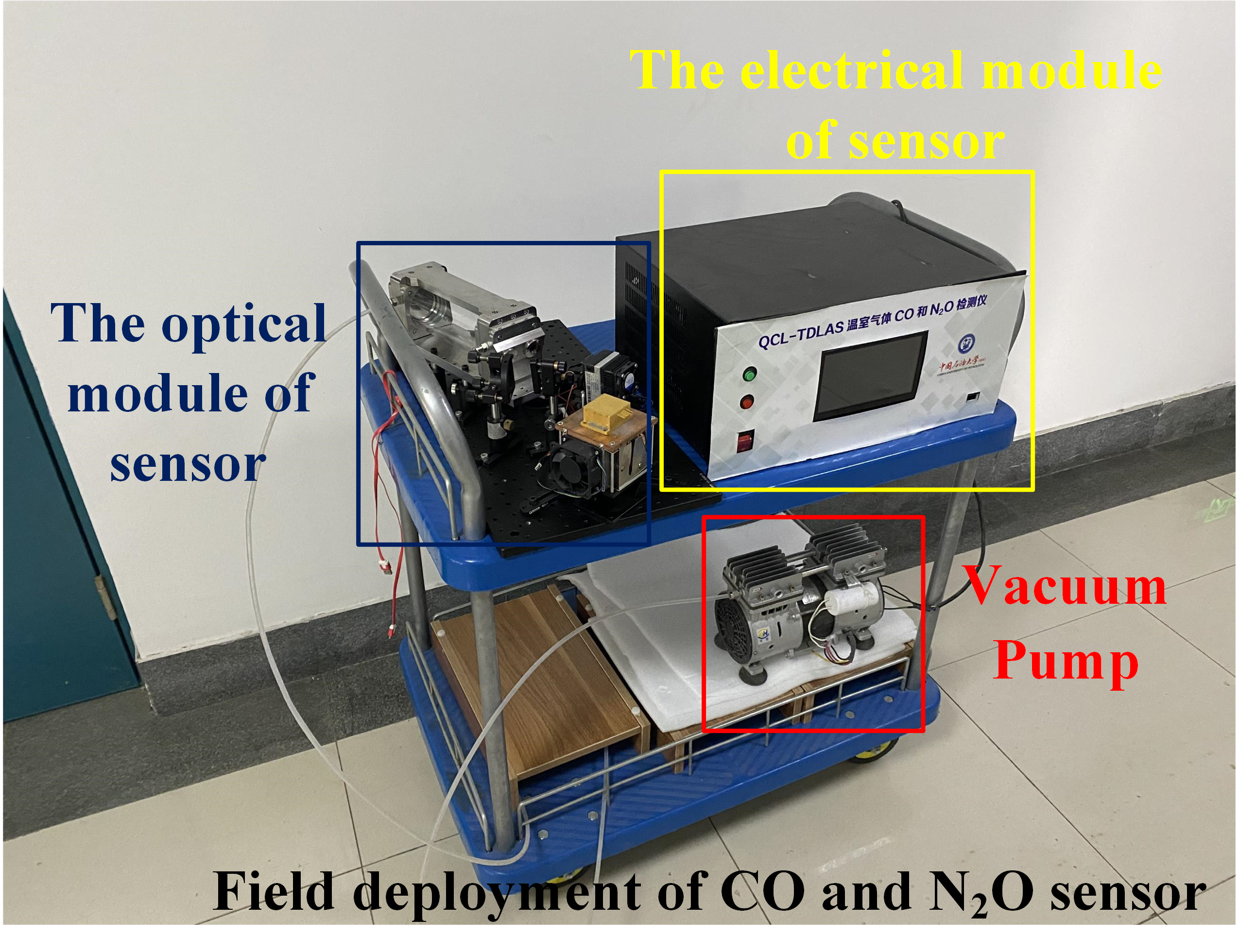


**Supplementary Figure 9.** **(a)** Photograph of the sensor for the field deployment.





**Supplementary Figure 9.** **(b)** The real-time measured concentrations of the sensor.

## Supplementary Tables

**Supplementary Table 1** Comparison of fitting precision of BP neural network, PLS and PSO-KELM algorithms for step test of N_2_O gas.

| Algorithm | RMSE/ppb | *R^2^* |
| --- | --- | --- |
| PSO-KELM | 11.688 | 0.9997 |
| BP neural network | 36.757 | 0.9975 |
| PLS | 60.078 | 0.9683 |
